# Immunohistochemical profiles of claudin-3 in primary and metastatic prostatic adenocarcinoma

**DOI:** 10.1186/1746-1596-6-12

**Published:** 2011-01-21

**Authors:** Tanner L Bartholow, Uma R Chandran, Michael J Becich, Anil V Parwani

**Affiliations:** 1 University of Pittsburgh School of Medicine, Pittsburgh, PA, USA; 2 Department of Biomedical Informatics, University of Pittsburgh School of Medicine, Pittsburgh, PA, USA; 3 Department of Pathology, University of Pittsburgh School of Medicine, Pittsburgh, PA, USA

## Abstract

**Background:**

Claudins are integral membrane proteins that are involved in forming cellular tight junctions. One member of the claudin family, claudin-3, has been shown to be overexpressed in breast, ovarian, and pancreatic cancer. Here we use immunohistochemistry to evaluate its expression in benign prostatic hyperplasia (BPH), prostatic intraepithelial neoplasia (PIN), normal tissue adjacent to prostatic adenocarcinoma (NAC), primary prostatic adenocarcinoma (PCa), and metastatic prostatic adenocarcinoma (Mets).

**Methods:**

Tissue microarrays were immunohistochemically stained for claudin-3, with the staining intensities subsequently quantified and statistically analyzed using a one-way ANOVA with subsequent Tukey tests for multiple comparisons or a nonparametric equivalent. Fifty-three cases of NAC, 17 cases of BPH, 35 cases of PIN, 107 cases of PCa, and 55 cases of Mets were analyzed in the microarrays.

**Results:**

PCa and Mets had the highest absolute staining for claudin-3. Both had significantly higher staining than BPH (p < 0.05 in both cases) and NAC (p < 0.05 in both cases). PIN had a lower, but non-significant, staining score than PCa and Mets, but a statistically higher score than both BPH and NAC (p < 0.05 for both cases). No significant differences were observed between PCa, Mets, and PIN.

**Conclusions:**

To our knowledge, this represents one of the first studies comparing the immunohistochemical profiles of claudin-3 in PCa and NAC to specimens of PIN, BPH, and Mets. These findings provide further evidence that claudin-3 may serve as an important biomarker for prostate cancer, both primary and metastatic, but does not provide evidence that claudin-3 can be used to predict risk of metastasis.

## Background

During 2010 alone, 32,050 deaths attributable to prostate cancer are expected to occur in the United States, making it the second leading cause of cancer death in males[1]. Despite this, studies conducted before the era of prostate specific antigen (PSA) screening have shown that latent prostate cancer was often diagnosed only after autopsy in older males[2-4]. One autopsy study of males over the age of 50, also conducted before PSA screening was implemented, indicated that the overall age-adjusted prevalence of latent prostate carcinoma was as high as 34.6% for whites and 36.9% for blacks in the United States[5], providing evidence that not all cases of prostate cancer have the same clinical aggressiveness. Based upon cases of prostate cancer diagnosed by PSA, researchers have estimated that clinically insignificant prostate cancer is now actually overdiagnosed at a rate of 29% for whites and 44% for blacks, the PSA screen resulting in the detection of cancers that otherwise would only have been detected during autopsy in up to 15% and 37% of tumors in whites and blacks, respectively[6].

There is limited information in the literature on predictive biomarkers to discern which cases of prostate cancer are likely to remain latent, versus those that are likely to metastasize and warrant more aggressive management[7]. Understanding the protein expression patterns associated with prostate adenocarcinoma may provide a new toolset to aid in the diagnosis of prostate cancer, provide prognostic information about the risk of metastasis, and indicate unique treatment targets.

Cellular tight junctions have been shown to play roles in maintaining cell polarity, regulating ion flow, and signalling[8-13]. Given that the disruption of tight junctions is believed to be one facet of tumorigenesis[14], claudins, one of the transmembrane protein families hypothesized to be the backbone of tight junctions[15], have become a target of focus in the recent cancer literature.

One specific member of this family, claudin-3, has been shown to be overexpressed in multiple forms of cancer, having been most thoroughly studied in ovarian cancer, where it has been shown to be up regulated more than 80-fold in comparison to normal ovarian cells[16]. In ovarian serous adenocarcinoma, its increased expression has also been associated with shorter survival times[17]. Additional studies have shown that it is also up regulated in breast, pancreatic, bladder, thyroid, fallopian tube, ovary, stomach, colon, and uterine cancer[18]. Moreover, it has been observed in 18% of mesotheliomas and 90% of cases of metastatic lung adenocarcinoma[19]. A significant inverse relationship between claudin-3 expression and overall survival in renal clear cell carcinoma has been documented[20].

In prostate cancer specifically, claudin-3 has been shown to correlate with advanced tumor stage and recurrence, although the immunohistochemical expression profiles varied in an examination of 141 cases of prostate cancer, where matched adjacent normal epithelium was compared to prostatic adenocarcinoma (PCa). In 31% of cases, the expression was actually lower than in the matched PCa [21].

Here, we compare the immunohistochemical profiles in a series of 53 cases of normal tissue adjacent to prostatic adenocarcinoma (NAC), 17 cases of benign prostatic hyperplasia (BPH), 35 cases of prostatic intraepithelial neoplasia (PIN), 107 cases of PCa, and 55 cases of metastatic prostatic adenocarcinoma (Mets) in order to obtain a more complete understanding of the immunostaining profile of claudin-3 in primary and metastatic prostate cancer and to further examine its potential as a biomarker for prostate cancer and/or prostate cancer metastasis.

## Methods

### Preparation of Tissue Microarray Blocks

Two sets of tissue microarray (TMA) blocks were constructed using specimens located in the Health Sciences Tissue Bank at the University of Pittsburgh Medical Center. Cores were taken from the appropriate case specific paraffin-embedded tissue blocks and assembled into TMAs according to a previously described protocol[22]. The final TMAs consisted of 53 cases of NAC, 17 cases of BPH, 35 cases of PIN, 107 cases of PCa, and 55 cases of Mets. Each case was represented at least in triplicate, however, in some cases, due to processing, only two cores for each case were represented. In these instances, the cases were still included in the analysis.

### Immunohistochemistry

Each TMA block was deparaffinized and then rehydrated with incremental ethanol concentrations. Decloaker was then used for heat induced epitope retrieval, followed by a 5 minute TBS buffer rinse. A Dako Autostainer was then used to stain the TMAs with anti-claudin-3 (working dilution 1:150), a rabbit polyclonal antibody (Catalogue # RB-9251-PO) from Thermo Scientific (Waltham, MA, USA). Immunolabeling was conducted using Rabbit Envision + HRP Polymer (Catalogue # K4003) from Dako (Carpinteria, CA). Slides were counterstained with hematoxylin before being coverslipped.

### Scoring of Slides

Slides were scored on a scale of staining intensity, with 0 representing no staining, 1 representing weak staining, 2 representing moderate staining, and 3 representing strong staining. Each staining value was multiplied by the percentage of the core that stained for that intensity, expressed as a whole number. For cores where more than one intensity was represented, the sum of the staining values was calculated. An average value was obtained for each case, and subsequently, each tissue type. This scoring procedure was modified from a scoring protocol previously used by Parwani, et al[23].

Means by Gleason score and tumor stage, where available, were also obtained. All means were reported with standard error. A one-way ANOVA with subsequent Tukey tests for multiple comparisons or nonparametric Kruskal-Wallis test with post-hoc Dunn's tests (α = 0.05 in both cases) were used to compare the tissue types, PCa carcinoma stages, and PCa Gleason scores.

Photomicrographs of tissue cores were taken using an Olympus BX51 microscope using Spot Advanced V4.6 (Diagnostic Instruments, Inc.) software. All images were taken at 20x.

This study received exempt approval (PRO08040368) from the University of Pittsburgh Institutional Review Board.

## Results

### Staining Intensities

**Figure 1:**
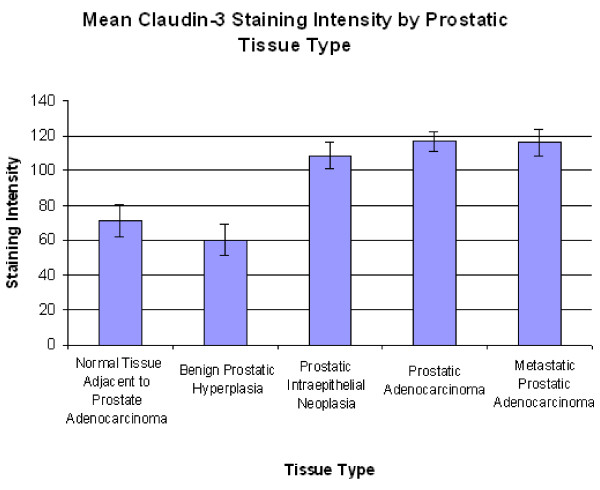
**Mean claudin-3 staining score by prostatic tissue type**. Mean claudin-3 staining score by prostatic tissue type. Significant differences were seen between NAC and PIN, NAC and PCa, NAC and Mets, BPH and PIN, BPH and PCa, and BPH and Mets (p < 0.05 for each). No differences were seen between PCa, Mets, and PIN.

The mean staining scores for NAC, BPH, PIN, PCa, and Mets were 71.42 ± 8.78, 60.23 ± 9.42, 108.76 ± 7.67, 117.03 ± 5.38, and 116 ± 7.66 (Figure 1).

A Kruskal-Wallis test (p = < 0.001), with subsequent Dunn's tests showed significant differences between NAC and PCa, BPH and PCa, NAC and Mets, BPH and Mets, NAC and PIN, and BPH and PIN (p < 0.05 in all cases). No significant differences were seen between PCa and Mets or PCa and PIN (p > 0.05 in both cases). Twelve of 53 cases (23%) of NAC, 2 of 17 cases (12%) of BPH, 20 of 35 cases (57%) of PIN, 64 of 107 cases (60%) of PCa, and 33 of 55 cases (60%) of Mets had staining scores higher than 100.

**Figure 2:**
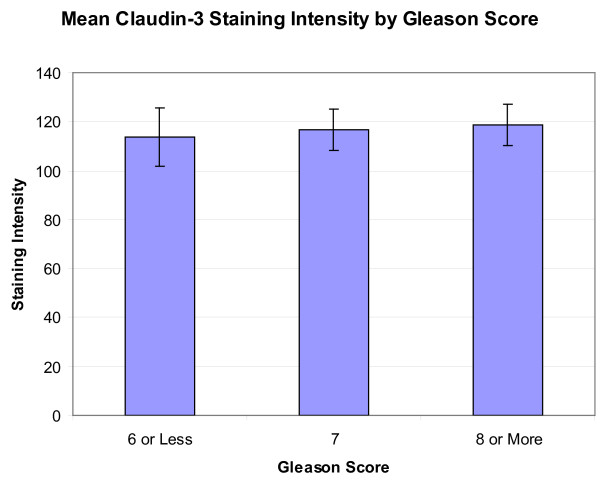
**Mean claudin-3 staining score by Gleason score**. Mean claudin-3 staining score by PCa Gleason score. No significant differences were seen by this classification (P = 0.950).

When classified by Gleason score, the average staining score was 113.63 ± 12.08 (n = 17) for those with a score of 6 or less, 116.81 ± 8.42 (n = 51) for those with a score of 7, and 118.80 ± 8.53 (n = 39) for those with a score of 8 or more (Figure 2). A resultant one-way ANOVA showed no significant differences (p = 0.9504). Twelve of 17 cases (71%) with a Gleason score of 6 or less, 29 of 51 cases (57%) with a Gleason score of 7, and 24 of 39 cases (62%) with a Gleason score of 8 or higher had staining scores higher than 100.

**Figure 3:**
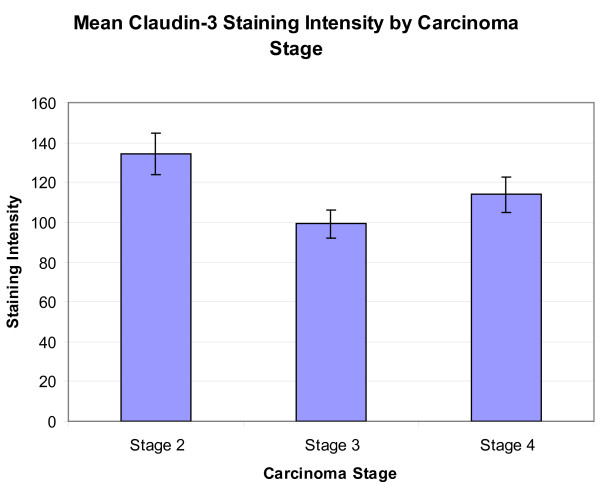
**Mean claudin-3 staining score by carcinoma stage**. Mean claudin-3 staining score by PCa carcinoma stage. Significant differences were seen between stage 2 and stage 3 (p = 0.010).

When classified by tumor stage, the mean scores were stage 2, 134.34 ± 10.25 (n = 39), stage 3, 98.25 ± 6.91 (n = 38), and stage 4, 113.95 ± 8.88 (n = 29) (Figure 3). A resultant one-way ANOVA (p = 0.013) with subsequent Tukey tests for multiple comparisons showed significant differences between stage 2 and stage 3 (p = 0.010). Twenty-eight of 39 cases (72%) of stage 2 carcinoma, 18 of 38 cases (47%) of stage 3 carcinoma, and 18 of 29 cases (62%) of stage 4 carcinoma had staining scores higher than 100.

**Figure 4:**
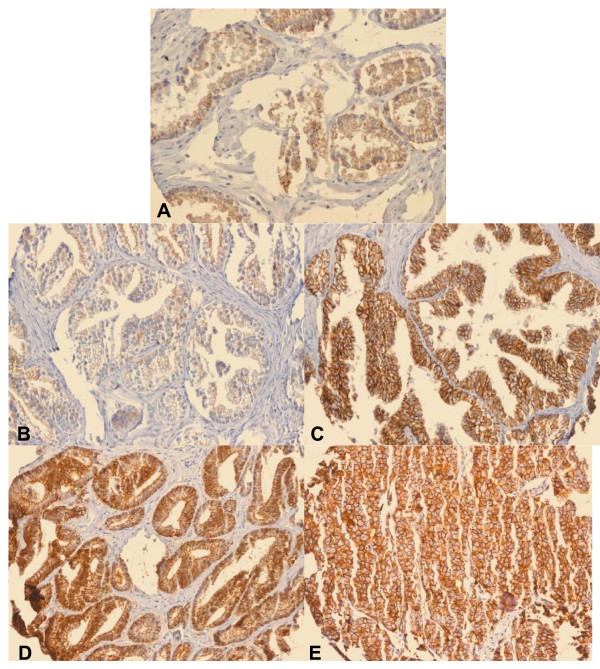
**Photomicrographs of TMA cores**. Photomicrographs of TMA cores (20x). A) NAC, B) BPH, C) PIN, D) PCA, and E) Mets. Average staining was higher in PCa and Mets than in NAC and BPH. The immunostain was predominantly membranous in positive cases, however cytoplasmic staining was additionally noted in select cores as depicted most prominently here in the photomicrographs of PCa and Mets.

Representative photomicrographs of the TMA cores are shown in Figure 4. Claudin-3 staining was a predominantly membranous in positive cases, although in some cores, depicted most prominently in the primary and metastatic photomicrographs, additional cytoplasmic staining was also noted.

## Discussion

In the claudin-3 stained specimens, the average staining scores were highest in PCa and Mets. PIN had a lower absolute staining score than PCa and Mets, although the differences were not significant. Both BPH and NAC had significantly less staining than PCa and Mets (Figure 1). These differences support the notion that claudin-3 is upregulated in many cases of prostatic adenocarcinoma. It does not appear that stronger expression exists in all cases, however, as there were 42 cases of PCa and 22 cases of Mets that had staining scores below 100.

Previous studies have shown that Gleason scores of 7 or higher were more frequently associated with lower expression of claudin-1[24]. Additionally, Landers, et al., demonstrated that claudin-4 was upregulated in primary and metastatic prostate cancer, although they state that it tended to be expressed more in primary tumors with a Gleason score of 6 than those with a score of 7 or higher[25]. In this current study, no significant differences were seen when the cases of PCa were compared by Gleason score (Figure 2).

Sheehan, et al., have also reported that claudin-3 expression correlates with advanced stage and tumor recurrence in prostate cancer [21], which coincides with another study of urothelial carcinoma, where claudin-3 has also been shown to correlate with advanced stage and poor survival[26]. In this current study, however, the highest average staining appeared to be in stage 2 PCa, which was significantly higher than stage 3 PCa staining, but not significantly higher than that noted in stage 4 PCa (Figure 3). Although statistically different, it is worth noting that these absolute staining scores are similar, reflecting that the difference in stage staining may not be of enough magnitude to use clinically. In a study of gastric cancer, claudin-3 has also been shown to be less expressed in advanced stage cases[27], which may suggest that claudin family expression patterns vary by cancer type.

As claudin-3 is a tight junction protein, it is interesting to note that in addition to membranous staining, cytoplasmic staining was also seen in select cores, most prominently in cases of PCa and Mets (Figure 4). Rangel, et al., have reported positive cytoplasmic immunostaining for claudin-3 in ovarian tumors and have suggested that this mislocation may be the result of abnormal pathway activation in cancer[28]. This finding may indicate that a similar occurrence takes place in prostate cancer and may warrant further investigation.

Based on this study, it appears that claudin-3 is moderately to strongly expressed in the majority of cases of prostate cancer and may serve as an important biomarker for prostate cancer diagnosis, both primary and metastatic, however the discrimination between primary and metastatic cases in this study is not great enough to merit its usage as a marker to predict the risk of metastasis.

Some groups have speculated on the specific mechanism whereby claudin-3 may be involved in carcinogenesis. D'Souza, et al., have shown that tight junction strength decreased when claudin-3, when designed to contain a T192D mutation mimicking a phosphorylated state, was overexpressed in ovarian cancer cell line OVCA433, a mechanism possibly enabling invasion[29]. Additionally, Agarwal, et al, have noted that ovarian epithelial cells specifically designed to constitutively express claudin-3 were found to have higher activity of matrix metalloproteinase-2, which may also contribute to invasion, although this level did not decrease following siRNA knockdown of claudin-3, possibly indicating that other pathways also promote its expression[30]. This could be one explanation for the fact that strong claudin-3 staining is not noted in all cases of PCa.

Finally, in addition to its potential role as a predictive biomarker for cancer, claudin-3 has also been shown to bind clostridium perfringens enterotoxin, subsequently leading to toxin-mediated cytolysis, prompting some to suggest that this may indicate a future therapy in select cases of prostate cancer[31,32]. Should such a therapy be developed, the immunohistochemical profiles of patients for this marker may become even more important, as it may serve to guide therapy selection.

## Conclusions

These results provide a basis for the characterization of claudin-3 staining in both primary prostatic adenocarcinoma and metastatic prostatic adenocarcinoma. In both types of tissue, increased expression of claudin-3 was seen in the majority of cases examined, and was seen more frequently than in non-neoplastic tissue, indicating that it may serve as an important biomarker for prostate cancer. Despite this, the differences in primary and metastatic cancers were not enough to indicate that a claudin-3 immunostain could provide prognostic information about the risk of metastasis. Furthermore, no significant differences were seen when the cases of PCa were compared by Gleason score. While stage 2 mean staining was significantly greater than stage 3, the numerical differences were similar in absolute. Given its high expression in the majority of cases of prostate cancer, and the fact that it has been implicated as a possible therapeutic target, claudin-3 warrants additional studies to evaluate its potential as a clinically useful biomarker in the diagnosis of prostate cancer.

## Competing interests

The authors declare that they have no competing interests.

## Authors' contributions

TB assisted in scoring tissue microarrays under the direct supervision of an attending pathologist, performed the statistical calculations, and drafted the manuscript. UC assisted with statistical calculations and reviewed the manuscript. AP conceived of the study, developed and approved the study protocol, approved all tissue microarray scoring, and revised the manuscript. MB also conceived of the study, developed and approved the protocol, and revised the manuscript. All authors have read and approved the final manuscript.
